# Static and Dynamic Prediction of Chronic Renal Disease Progression Using Longitudinal Clinical Data from Taiwan’s National Prevention Programs

**DOI:** 10.3390/jcm10143085

**Published:** 2021-07-13

**Authors:** Yi-Ping Chang, Chen-Mao Liao, Li-Hsin Wang, Hsiu-Hua Hu, Chih-Ming Lin

**Affiliations:** 1 Division of Nephrology, Taoyuan Branch of Taipei Veterans General Hospital, Taoyuan 330, Taiwan; ypchangtyvh@gmail.com; 2 Department of Healthcare Information and Management, Ming Chuan University, No. 5, De Ming Rd., Gui Shan District, Taoyuan 333, Taiwan; 3 Department of International Business, Ming Chuan University, Taipei 111, Taiwan; shhu@mail.mcu.edu.tw; 4 Department of Applied Statistics and Information Science, Ming Chuan University, Taoyuan 333, Taiwan; cmliao@mail.mcu.edu.tw (C.-M.L.); wang.lihsin4@gmail.com (L.-H.W.)

**Keywords:** chronic kidney disease, dialysis, glomerular filtration rate, urine protein, creatinine

## Abstract

Kidney diseases can cause severe morbidity, mortality, and health burden. Determining the risk factors associated with kidney damage and deterioration has become a priority for the prevention and treatment of kidney disease. This study followed 1042 chronic kidney disease (CKD) patients with Stage 3–5 kidney disease who were treated at a public veteran’s hospital through the national prevention program. A total of 12.5 years of records of clinical measurements were collected and analyzed using dynamic and static Cox hazard models to predict the progression to dialysis treatment. The results showed that the statistical significance of several variables in patients with Stage 3–5 CKD was attenuated while the dynamic model was being used. The estimated glomerular filtration rate (eGFR) and urine protein to creatinine ratio (PCR) had the powerful ability to predict the progression of CKD patients with Stage 3a and Stage 3b–5 kidney disease, whereas serum calcium was also predictive for the progression of Stages 3b–5 CKD. Because these two sub-stages of Stage 3 CKD are often associated with differences in routine measurements and the risk analysis of renal dialysis, future research can use this predictive model as a reference while similar prevention programs are implemented.

## 1. Introduction

Kidney disease has been ranked among the top 10 causes of death in Taiwan since 1990. There were 5096 deaths due to nephritis, nephrotic syndrome, and nephrosis in 2020, which resulted in a mortality rate of 21.6 per 100,000 [1]. According to a database maintained by a large standard medical screening program, the prevalence of chronic kidney disease (CKD) in Taiwan is 11.9%, and the increase in end-stage renal disease (ESRD) has been associated with an increase in the incidence of diabetic nephropathy [2]. Although Taiwan has a lower prevalence rate of CKD than the United States (15%) [3] and Japan (13%) [4], the ESRD rate is higher in Taiwan than in those other countries. According to the United States Registration Data System (USRDS), the prevalence rate of ESRD in Taiwan was 3587 per million people in 2018, ranking first in the world [5].

Dialysis costs for ESRD patients have been fully reimbursed by the National Health Insurance (NHI) program in Taiwan since 1995. More than 90,000 patients received dialysis therapy (hemodialysis and peritoneal dialysis) in 2018, and the annual expenditure for dialysis therapy has been estimated at 1.5 billion USD in Taiwan [6]. ESRD has become the single disease associated with the highest annual health insurance cost. Because the cost associated with ESRD treatments is expensive, ESRD has become a heavy burden on NHI finances; therefore, identifying risk factors associated with kidney damage and deterioration has become a priority for the prevention and treatment of kidney disease.

Currently, the clinical management and treatment of patients with CKD are based on CKD severity or staging. The severity and need for nephrology referral are primarily based on the estimated glomerular filtration rate (eGFR) of serum creatinine. However, previous studies have suggested that eGFR alone is an insufficient measure for clinical decision-making [7,8]. The ideal disease prediction model should be correct, easy to operate, and applicable to patients with different forms of kidney disease and who belong to different ethnic groups. The kidney failure prediction model developed by Tangri et al. in 2011 is currently the most widely applied method. Tangri et al. used data from two separate CKD cohorts in Canada, including Stage 3 to Stage 5 patients, to develop and validate risk-predictive models for CKD progression. The results showed that a lower eGFR value is an important factor that can predict faster progression to kidney failure [9]. This model was also developed into a kidney failure risk equation (KFRE), which provides a score estimation with clinical value. Another study also showed that a lower eGFR value was associated with a gradual increase in the risk of CKD progression to ESRD [10]. In addition, higher levels of proteinuria, old age, and male sex were also found to accelerate renal function decline, and a higher risk of CKD progression was observed in patients with proteinuria and high-protein diets [11,12]. Chang et al. retrospectively reviewed and analyzed 1549 CKD patients who participated in the “Public health insurance Pre-ESRD preventive program and patient health education program” to establish an optimal prediction model for CKD progression. They found that the influencing factors with the best explanatory power included age, serum creatinine (Cr), urea nitrogen, and comorbidity with diabetes [13].

Although many previous studies have attempted to define the best model for predicting CKD progression, one limitation has been that their databases were ”static” and did not consider previous values or track follow-up developments. Tangri et al. conducted a “dynamic” predictive model using demographic, clinical, and time-dependent laboratory data from 3004 patients with Stage 3 to 5 CKD in Canada [14]. They used the latest-available measurement model with time-dependent predictors and compared this model with the baseline model. The results showed that eGFR remained strongly associated with renal failure in the dynamic model compared with the baseline static model, but other variables, such as male sex, serum phosphorus, albumin (ALB), and bicarbonate levels, were no longer significant. They concluded that a dynamic predictive model for CKD progression might improve risk prediction over a static model.

To promote the prevention and treatment of chronic kidney disease, Taiwan established the program “NHI Pre-ESRD patient care and education program” for patients with Stage 3b–5 CKD in 2006, and the program “NHI reimbursement plans that improve health care quality of early-stage chronic kidney disease” for Stage 1–3a CKD in 2011, which are administered by the *National Health Insurance Administration* (NHIA), Ministry of Health and Welfare [15]. The goals of both programs are to decrease the incidence of ESRD in addition to decreasing the morbidity and mortality of CKD. Simultaneously, these programs aimed to prevent, reduce, and delay the rate of renal function decline and to improve the quality of care. However, the follow-up procedures between these two programs differ in terms of the biochemistry tests performed. The early-stage CKD disease management program performs follow-up for the urine protein to creatinine ratio (PCR), serum Cr, and low-density lipoprotein (LDL), with the addition of glycated hemoglobin (HbA1c) among diabetic patients every 6 months. In contrast, the pre-ESRD CKD disease management program performs follow-up for hemoglobin (Hgb), blood urea nitrogen (BUN), serum Cr, and ALB every 3 months; serum total calcium (Ca), phosphate (P), fasting glucose, and HbA1c every 6 months; and serum LDL, uric acid, sodium (Na), potassium (K), triglyceride (TG), and urine PCR every 12 months.

Static modeling, previously developed, is beneficial even for early disease prevention, while the dynamic modeling infers association by considering time-varying covariates. The aims of this study were to develop both static and dynamic modeling to predict the risk factors for eventual dialysis treatment using the available clinical data that are routinely measured in CKD patients. Simultaneously, we aimed to evaluate the value of the dialysis prediction model for two CKD disease management programs in Taiwan.

## 2. Methods

### 2.1. Study Design and Patient Population

**Figure 1:**
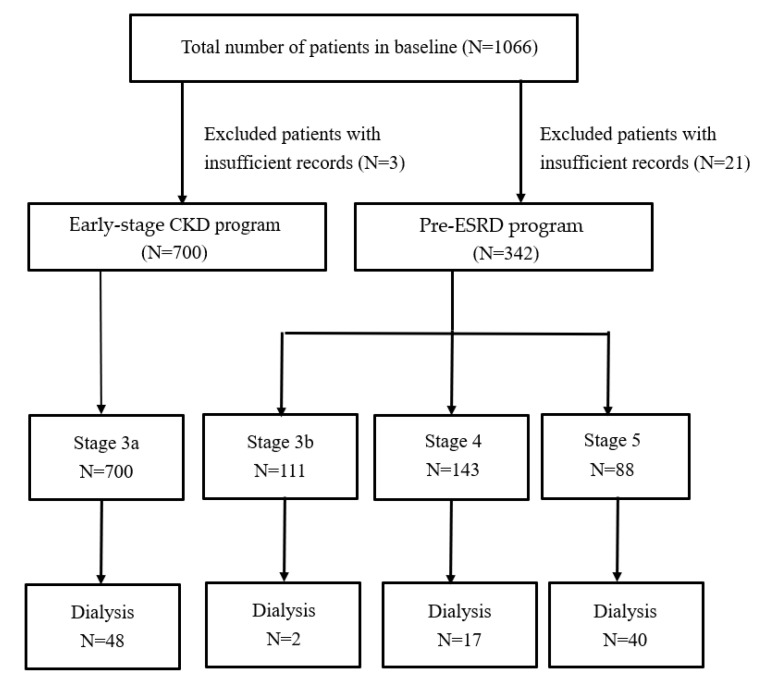
Flow chart for the selection of the study subjects.

This study was designed as a retrospective cohort study, in which data from the “NHI Pre-ESRD patient care and education program” and “NHI reimbursement plans that improve health care quality of early-stage chronic kidney disease” programs implemented by the *National Health Insurance Administration* (NHIA) were examined. This study enrolled patients treated at the Taoyuan Branch of Taipei Veterans General Hospital from January 2006 to July 2019. De-identified data of patients with Stage 3 to 5 (eGFR < 60 mL/min/1.73 m^2^) CKD were reviewed. CKD patients were defined as those who presented to the hospital for the first time for CKD treatment and then returned for follow-up visit at intervals of at least every 6 months and who were diagnosed with CKD according to the related International Classification of Disease, 9th Revision Clinical Modification (ICD-9-CM) codes (shown in Table S1) or ICD-10 codes. The follow up period, which included blood tests, physical measurement, and CKD education, was every 6 months for patients in the early CKD program and, at a minimum, every 3 months for pre-ESRD program patients until the start of dialysis therapy initiation or of kidney transplantation up to 31 July 2019. Patients who were transferred to other hospitals, died, or were censored for other reasons during the following period of time were defined as an endpoint of observation without ESRD. Hazard of developing ESRD was estimated for each 90-day period starting from a patient’s first nephrology clinic visit until the maximum follow-up time. The latest record was defined as a laboratory test requisition in patients without death before the endpoint date. After excluding 24 patients with insufficient records who had variables missing greater than 30% of values, the remaining subjects were stratified by the two NHIA programs. In total, 1042 subjects, comprising 700 patients with Stage 3a CKD, 111 with Stage 3b, 143 with Stage 4, and 88 with Stage 5, were included in this study. The number of patients with the four CKD stages who had dialysis therapy initiation during the study period were 48, 2, 17, and 40, respectively. A flow chart of the data collection process used to identify study subjects is shown in Figure 1.

### 2.2. Variables

The outcome in this study was ESRD, defined as the initiation of hemodialysis or peritoneal dialysis. There was no patient treated by kidney transplantation in our study subjects. As the follow-up procedures between these two NHIA programs differ in terms of the biochemistry testing, blood tests were carried out during the clinic visit according to procedures for each enrolled patient, and were followed up every 6 months (Stage 3a) or at least every 3 months (Stages 3b–5) until death, the start of maintenance dialysis, or loss to follow-up. In the models, the clinical characteristics were classified into four main categories: demographic variables, including sex, age, height, and weight; laboratory data associated with CKD severity, assessed in both serum and urine, including eGFR, Hgb, hematocrit (Hct), serum ALB, Cr, and BUN levels, as well as Na, K, Ca, P, and urine PCR; comorbidity conditions, including hypertension, diabetes, and other cardiovascular diseases (CVDs); and other underlying risk-related biophysical and biochemical data, such as blood pressure, uric acid, lipid profile, fasting glucose, and HbA1c levels. All the baseline characteristics and laboratory variables were obtained from these two NHIA programs for analysis. According to the requirement of plans from NHIA programs, eGFR was calculated by using simplified Modification of Diet in Renal Disease (MDRD) equation, as follows [16]:

$$\mathrm{eGFR}=186\times {\mathrm{age}}^{-0.203}\times {\mathrm{Cr}}^{-1.154}\times \left(0.742,\;\mathrm{if}\;\mathrm{female}\right)$$

where age is the age of the patient (in years) and the extra coefficient of 0.742 should be multiplied if the patient is female. Based on previous literature, the four-variable MDRD study equation is the most widely used and showed greater accuracy for the calculation of creatinine clearance than the Cockcroft–Gault (CG) formula or Chronic Kidney Disease Epidemiology Collaboration (CKD-EPI) in patients with CKD, principally with Stage 3 to 5 CKD [17,18]. The PCR was log transformed due to its skewed distribution. Individual’s predictor variables were obtained initially from the first clinic visit during the study period. The baseline characteristics were defined as the test result in the first clinic visit. In dynamic analysis, to gain consistent frequency of records for each patient, only the latest individual’s measurement was included in analysis when multiple measurements for the same clinical characteristic were performed during each following 90-day period. Because different sets of laboratory data were available for the two NHIA programs, fewer clinical characteristics were analyzed in the four categories for patients with Stage 3a CKD. The missing parameters include Hgb, Hct, AlbB, BUN, Na, K, Ca, P, uric acid, cholesterol, triglyceride, and fasting glucose. Those variables missing greater than 30% of values were excluded from the analysis. The missing values for other variables were replaced with multiple imputation [19]. The study created five datasets using the multivariate imputation via chained equations (MICE) module in the R package to perform the data imputation.

### 2.3. Statistical Analysis

In this study, the baseline model used demographic and laboratory data from the first clinic visit, whereas the dynamic model used time-dependent covariates in survival analysis, similar to the approach used by Tangri et al. [14]. The Cox proportional hazard model was used to perform survival analysis to investigate the risks of CKD progression to dialysis. Univariate and multivariable hazards of developing kidney failure were estimated for each 90-day period, starting from a patient’s first nephrology clinic visit until the maximum follow-up time. Values from the previous period were carried forward if updated values were unavailable. Due to differences in the collection of clinical characteristics, data for patients with Stage 3a and Stage 3b–5 were processed separately. By applying univariate Cox proportional hazards regression models, variables not associated with renal failure (*p* > 0.10) were excluded from further multivariable analyses. In the multivariable analysis, four stratum models were developed by including the available variables, such as laboratory data associated with CKD severity, demographic data, comorbid conditions, and other biophysical and biochemical data associated with underlying risk. According to these inclusion criterions, two and four available stratum models were developed and compared for patients with Stage 3a and Stage 3b to 5 CKD, respectively. Improvements in model performance were examined through the addition of new candidate variables models by performing multivariate Cox proportional hazards regression. The optimal model was selected by the significance for the difference of likelihood ratios when the likelihood ratio was compared with that in the previous model. The presence of collinearity was examined using a correlation matrix, followed by the evaluation of variance inflation factors and the magnitude of standard errors. In the baseline model, we included all covariates as time-independent variables in a Cox proportional hazard model. In the dynamic model, values for the dynamic covariates that were updated at each clinic visit were used and modeled using the time-dependent module in the R package created by Therneau et al. [20]. Values from the previous period were carried forward if updated values were unavailable. We included all measurements except sex as time-dependent covariates for CKD patients categorized as Stage 3a. Due to fewer than three PCR measurements being collected from each patient categorized as Stage 3b–5, sex and urinary PCR were treated as time-independent covariates, using their baseline values. We included age and other laboratory variables as time-dependent covariates, using values from all visits. The dynamic hazards model can be expressed in the following function:

$$\lambda \left(t\right)=\lambda_{0}\left(t\right)e^{\beta Z\left(t\right)}$$

where $\lambda_{0}\left(t\right)$ is the baseline hazard function, λ(t) denotes the time-dependent hazard function, $\beta =\left(\beta_{1},\ldots ,\beta_{p}\right)$ is the coefficient of covariance, and $Z\left(t\right)=\left(Z_{1}\left(t\right),Z_{2}\left(t\right),\ldots \ldots ,Z_{p}\left(t\right)\right)$ is covariance at time *t*. The Akaike information criterion and Harrell’s C statistics were computed as measures of model discrimination. Two and four available classified models were developed and compared for patients with Stage 3a and Stage 3b to 5 CKD, respectively. The optimal model was selected by the significance for the difference of likelihood ratios. Kaplan–Meier plots were generated to evaluate the survival functions of the follow-up data. A variance inflation factor (VIF) was used to discriminate the collinearity of the models. The Schoenfeld residual test for a non-zero slope assumption was performed as well. All analyses and figure creation were performed with R version 3.2.5 (R Foundation for Statistics Computing, Vienna, Austria).

### 2.4. Ethical Approval

The study was in accordance with relevant guidelines and regulation. The study protocol used was reviewed and approved by the Institutional Review Board of Taipei Veterans General Hospital (No. 2020-01-024BC). Because data were de-identified, the Institutional Review Board provided the waiver for the need of informed consent for the retrospective study.

## 3. Results

After excluding 24 patients with records missing variables that were missing greater than 30% of values, the study included 1042 patients with Stage 3 to 5 CKD who were included in the final analysis. The missing values for other variables were replaced using the multiple imputation method. Table 1 shows the demographic data for the sample population included in this study according to the CKD stage. Approximately two-thirds of patients (67.2%) were classified as Stage 3a CKD at baseline, defined as the time of program enrollment. A majority of patients were older than 80 years (79.8 ± 0.8 years), and men accounted for 69.3% of the study population. The average eGFR among Stage 3a patients was 51.3 mL/min/1.73 m^2^, compared with 37.1 mL/min/1.73 m^2^ for Stage 3b patients. The urine PCR value nearly doubled between Stages 3a and 3b (448.8 vs. 987.2, respectively, on average). The average number of measurements obtained during follow-up was 3.3, and the average duration of follow-up was 637.6 days (i.e., 1.75 years). Shorter duration of follow-up was observed in patients with later-stage CKD. A total of 107 patients received dialysis therapy (10.3%), and the proportion of patients with Stage 3a (6.9%) who progressed to dialysis treatment was higher than that of Stage 3b patients (1.8%). Diabetes and CVDs were more prevalent among Stage 3a patients than Stage 3b patients. 

For the baseline demographic and biochemical variables, we used univariate analysis to identify potential risk factors associated with dialysis treatment. Table 2 shows the clinical indicators, including age, eGFR, Hgb, serum ALB, BUN, Cr, Na, Ca, P, TG, urine PCR, and HbA1c, which were associated with a significant risk of dialysis among patients with Stage 3b–5 CKD. In contrast, the indicators associated with significant risk for dialysis treatment among Stage 3a CKD patients were eGFR, serum Cr, urine PCR, and HbA1c levels.

In the multivariate analysis, all cases were entered into the dialysis risk prediction model after excluding risk factors that were not identified as significant in the univariate analysis, and the best model was selected based on relevant statistics and likelihood ratio tests. Table 3 presents the adjusted hazard ratios (AHRs) for variables associated with the risk of dialysis treatment according to the “static” baseline model. Based on the significant differences in the likelihood ratios of the developed models, Model 1 for Stage 3a and Model 2 for Stages 3b–5 were selected as the optimal models. For patients with Stage 3a CKD, for every 1 mL/min/1.73 m^2^ increase in eGFR, the risk of renal dialysis can be reduced by 6%. However, for every 1-unit increase in the ln urine PCR value, the risk of renal dialysis may increase to 1.6-fold (Model 1). For patients with Stage 3b–5 CKD (Model 2), the risk-reducing factors included age (risk decreased by 3% for every 1-year increase), eGFR (the risk decreased by 7% for every 1-unit increase), and serum ALB (the risk decreased by 51% for every 1 g/dL increase). The risk-increasing factors were serum Cr and ln PCR, and every 1-unit increase in these variables increased the risk of renal dialysis by 9% and 49%, respectively. In the optimal baseline model, the collinearity analysis showed the models were appropriate (Table S2). The Schoenfeld residual test indicated that the models did not perform against the proportional hazards’ assumption (Figures S1 and S2).

**Figure 2:**
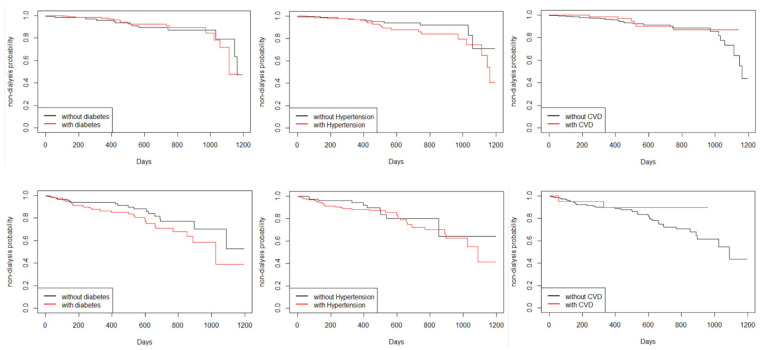
The Kaplan–Meier curves of non-dialysis for CKD patients with or without diabetes, hypertension, and cardiovascular disease in Stage 3a CKD (**upper panel**) and Stage 3b to Stage 5 CKD (**lowest panel**).

In this study, the *Kaplan–Meier* estimation method was used to analyze the impacts of comorbidities on the risk of dialysis. Figure 2 shows that patients with diabetes or hypertension together with Stage 3b–5 CKD have a relatively higher risk of renal dialysis after approximately 6 months than patients without comorbidities. For Stage 3a CKD, patients with comorbid hypertension showed an increased risk of renal dialysis after approximately 1.5 years.

The time-dependent dynamic prediction model was developed based on longitudinal data collected from patients with Stage 3 to 5 CKD (shown in Table 4). Model 1 was selected as the optimal model for both Stage 3a and Stages 3b–5 CKD. Among patients with Stage 3a CKD, eGFR and ln PCR remained the primary risk factors for dialysis treatment (Model 1). For each additional 1-unit increase, the risk of renal dialysis can be reduced by 7% for eGFR and increased by 72% for ln PCR. In addition to eGFR, the risk factors identified among patients with Stage 3b–5 CKD included Ca and baseline ln PCR. Each 1-unit increase reduced the risk of renal dialysis by 30% for Ca and increase the risk by 11% for ln PCR (Model 1).

## 4. Discussion

This study analyzed patients with Stage 3 to 5 CKD who participated in disease management programs for education and the prevention of CKD progression to ESRD in Taiwan. In this study, we found that the risk factors for the increased progression to dialysis treatment identified using the baseline CKD stage included reduced age, reduced eGFR, Hgb, serum ALB, serum Na, and serum Ca, as well as increased serum Cr, BUN, serum P, TG, urine PCR, and HbA1c level. These risk factors may accelerate renal function deterioration and enhance progression to dialysis treatment. These results are in line with those from previous studies [13,14,21].

Age was identified as a risk factor for CKD progression, and our study found that older patients with Stage 3b–5 CKD may be at a lower risk of progressing to dialysis treatment than younger patients (HR = 0.971), which is a similar outcome as that reported by a previous study [21,22,23]. O’Hare et al. examined 209,622 US veterans with Stage 3 to 5 CKD who were followed up for a mean of 3.2 years. Their finding suggested that older patients had lower rates of dialysis but higher rates of death, and among patients older than 65 years, the risk for death appeared to exceed the risk for ESRD [23].

In the baseline models for both Stage 3a and Stage 3b–5 CKD, the predicted risk factors for dialysis treatment including eGFR and urine PCR. Past experimental and clinical research has shown that proteinuria plays an important role [24] and is a powerful and independent risk factor for CKD progression [25]. The MDRD study discovered that patients with higher baseline proteinuria experienced a relatively faster rate of eGFR decline [26]. The Chronic Renal Insufficiency Cohort (CRIC) study also revealed a reduced risk for CKD progression among diabetic populations without proteinuria compared with populations with proteinuria [27]. Our study supports this observation and found that for every 1-unit increase in the ln urine PCR value, the risk of renal dialysis increased 1.6-fold among Stage 3a CKD patients and 1.5-fold among Stage 3b–5 CKD patients.

In addition, serum ALB levels were a risk factor for CKD progression for Stage 3b–5 CKD patients (AHR = 0.507). For every 1 g/dL increase in serum ALB, the risk of dialysis treatment can be reduced by 49%. Previous research demonstrated that hypoalbuminemia was a risk factor associated with CKD progression among the pediatric population [28]. The CRIC study also reported a hazard ratio of 3.48 for patients in the lowest serum ALB quartile relative to those in the highest quartile [29]. Decreased serum ALB levels were significantly associated with eGFR decline, and hypoalbuminemia may be related to proteinuria or underlying inflammation [30].

When examining the association between major comorbidities and the risk of dialysis treatment, our study found that patients with Stage 3b–5 CKD and diabetes were at risk of needing dialysis within 6 months of enrollment. Patients with comorbid hypertension presented a similar pattern. These results were similar to those reported by another Taiwan study conducted by Lin et al. Their findings showed that during 42 months of follow-up, the probabilities of remaining dialysis free among patients with Stage 3b, 4, and 5 CKD without diabetes were 89.46%, 79.88%, and 55.68%, respectively, compared with probabilities of 84.65%, 55.68%, and 9.64% for patients with diabetes. An increased dialysis risk was also observed after approximately 6 months of follow-up among patients with Stage 4 and 5 CKD but not among patients with Stage 3 CKD [21].

In our study, the number of patients with comorbid CVDs in the study was small (19.2%), and the duration of follow-up was short (less than 500 days for most patients); therefore, comparing the risk of dialysis between patients with and without CVD was difficult. Although the risk for CVD is higher among individuals with CKD, CVD is frequently underdiagnosed and undertreated [31]. Past research has shown that when eGFR declines, the incidence and severity of hypertension increases [32]. Although CKD and hypertension are both independent risk factors for CVD, the risks of CVD morbidity and mortality increased when both exist together [31]. Studies have also found that among patients with Stage 3 or 4 CKD, the risk of death due to CVD is higher than the risk for ESRD progression [33,34].

In the dynamic model, eGFR and urine PCR remained primary risk factors associated with outcome among Stage 3a CKD patients. In contrast, among patients with Stage 3b–5 CKD, the risk factors associated with dialysis treatment were eGFR, serum Ca, and urine PCR. For patients with is Stage 3a and Stage 3b–5 CKD, eGFR and urine PCR remained predictive risk factors for dialysis treatment, and these results are consistent with those reported by Tangri [14]. Therefore, eGFR and urine PCR appear to represent the most important time-dependent variables associated with the risk of progressing to dialysis treatment. While comparing AHR in the dynamic model to that in the static model, several significant variables in patients with Stage 3 to 5 CKD attenuated the statistical significance, such as age, ALB, and Cr. It could be considered that later measurements are more associated with dialysis than older ones in a dynamic estimation. Thus, urine PCR in post-stage CKD may dominate the effects rather than ALB and Cr.

Among the potential biochemical variables, the present study found that high serum Ca was associated with a lower risk of dialysis treatment (HR = 0.741) among patients with Stage 3b–5 CKD, according to the univariate analysis (Table 2). In the dynamic model, the serum Ca level remained a protective factor associated with renal dialysis. For every 1 mg/dL increase in serum Ca, the risk of renal dialysis was reduced by 30% (Table 4). Janmaat et al. [35] analyzed 15,755 adults with Stage 3–5 CKD treated by a large healthcare system and concluded that lower baseline-corrected serum Ca was associated with more rapid CKD progression. These results are also consistent with a cohort study performed in Taiwan that included 2144 outpatients with Stage 3–4 CKD [21]. Lower serum Ca levels at baseline may be a manifestation of vitamin D deficiency, which may play a pathophysiological role in CKD progression [33], suggesting that vitamin D deficiency or insufficiency might accelerate the progression of CKD [36,37]. Our results reported an additional evidence that time-varied serum Ca is associated with the risk of dialysis.

A review of previous relevant literature revealed that many studies did not distinguish between Stages 3a and 3b among Stage 3 patients [9,13,21]. However, our study found that these two sub-stages were associated with differences in the risk analysis of renal dialysis, which can be used as a reference for future research.

Our study had some strengths relative to the previous literature. First, Tangri et al. [14] presented a dynamic predictive model for the progression of renal failure in a Western society and suggested further studies comparing static with dynamic predictive models and outcomes. The usefulness of the dynamic model is in inferring association by considering time-varying covariates. On the other hand, it is intuited that the last-available measurements are more associated with event occurrence than older ones. Thus, the dynamic model results might more likely reflect the association as more closed to event occurrence. Then, the results may not so useful for even earlier prevention. Besides studying in a different racial population, our research can provide additional information for different clinical purposes due to using both static and dynamic predictive models to assess the risk factors associated with CKD progression. Because Taiwan has been ranked as the country with the highest prevalence of ESRD, the effective prediction and prevention of renal function decline may have clinical relevance. A second strength is that our database was obtained from two national programs in Taiwan, the clinical information was relatively complete, and it was easier to track patients due to their participation in the national program. Moreover, our hospital is a veteran’s hospital, and veterans have a certain degree of loyalty to the veteran’s hospitals in Taiwan, which facilitated the acquisition of long-term outpatient information and laboratory examinations necessary to complete the “dynamic” follow-up.

However, our analysis also had several limitations that should be considered. First, the database for this study was obtained from a hospital; therefore, the extrapolation of these findings to other populations may be difficult. Second, because the attribute of our hospital is a veterans hospital, and outpatients are mainly veterans, the average age of patients in the present study was 80, which is approximately 10 years older than those included in the studies conducted by Chang et al. [13] and Tangri et al. [14]; therefore, the results of this study may be more applicable to the elderly. Third, intervariability may be a result due to instrumentation measurement differences and operator or test criteria. Caution should be taken during prediction to avoid increased uncertainty. Finally, though there was no death during Stage 3a, 29 deaths before ESRD were observed during Stages 3b to 5 in our study. Because the outcome was ESRD, not mortality, this endpoint may be impossible to observe due to a prior occurrence of other events (such as death from another cause). Future studies that perform an analysis of competing risks are suggested to ensure that the results are unbiased. Finally, our analyzed biochemical variables were obtained from NHI programs. Due to limited national insurance resources, some biochemical items were not routinely tracked every 90 days. Additional studies using a prospective cohort approach can collect more adequate variables to reflect real-world clinical conditions.

## 5. Conclusions

The study analyzed the longitudinal measurements of patients suffering from CKD using both static and dynamic models to predict the risk factors of progression to dialysis treatment. The findings add some evidence to the healthcare domain for different clinical purposes. In dynamic prediction, for Stage 3a CKD, eGFR and PCR had powerful predictive abilities; for Stage 3b to 5 CKD, besides eGFR and lower PCR, the serum Ca level plays a protective factor associated with renal dialysis. Additionally, because these two sub-stages of Stage 3 are associated with differences in the follow-up measurements and the risk analysis of renal dialysis, future research can use these models as references while similar prevention programs are developed and implemented.

## Figures and Tables

**Table 1:** Patient characteristics during the baseline stage of chronic kidney disease and dialysis treatment during the study period.

Variables	All *n* = 1042	Stage 3a *n* = 700	Stage 3b *n* = 111	Stage 4 *n* = 143	Stage 5 *n* = 88	*p*-Value
Height (cm)	161.7 ± 0.8	161.7 ± 0.6	161.9 ± 1.5	160.9 ± 1.5	163.0 ± 6.8	0.667
Weight (kg)	65.4 ± 0.8	66.1 ± 0.9	65.9 ± 2.2	64.4 ± 2.2	60.8 ± 2.7	0.002
Age (years)	79.8 ± 0.8	80.0 ± 0.9	79.2 ± 2.2	80.0 ± 2.4	78.5 ± 2.8	0.681
Male (%)	722 (69.3%)	495 (70.7%)	73 (65.8%)	103 (72%)	51 (58%)	0.068
eGFR (mL/min/1.73 m^2^)	42.4 ± 0.2	51.3 ± 0.4	37.1 ± 0.8	23.1 ± 0.7	10.3 ± 0.7	<0.001
Hemoglobin (g/dL)	–	–	12.3 ± 0.3	11.2 ± 0.3	9.4 ± 0.3	<0.001
Hematocrit (%)	–	–	37.1 ± 1.1	34.1 ± 0.8	29.0 ± 0.9	<0.001
Serum albumin (g/dL)	–	–	3.8 ± 0.1	3.6 ± 0.1	3.3 ± 0.1	<0.001
Serum creatinine (mg/dL)	1.9 ± 0.1	1.3 ± 0.1	1.8 ± 0.1	2.6 ± 0.1	6.1 ± 0.8	<0.001
Urea nitrogen (mg/dL)	–	–	29.9 ± 1.7	42.4 ± 2.5	76.1 ± 6.5	<0.001
Sodium (mEQ/L)	–	–	140.6 ± 0.7	139.9 ± 0.5	140.1 ± 0.9	0.373
Potassium (mEQ/L)	–	–	4.5 ± 0.1	4.7 ± 0.1	4.7 ± 0.2	0.213
Calcium (mg/dL)	–	–	9.3 ± 0.6	8.9 ± 0.1	8.5 ± 0.1	0.018
Phosphate (mg/dL)	–	–	3.7 ± 0.1	3.9 ± 0.1	4.9 ± 0.3	<0.001
Urine protein/creatinine	949.1 ± 127.2	448.8 ± 74.5	987.2 ± 331.7	2067.7 ± 602.9	3063.4 ± 650.2	<0.001
Systolic blood pressure (mmHg)	136.1 ± 1.2	134.6 ± 1.4	137.3 ± 3.9	139.4 ± 3.4	140.4 ± 4.5	0.004
Diastolic blood pressure (mmHg)	73.6 ± 0.9	73.4 ± 0.9	73.6 ± 2.6	74.1 ± 2.4	73.5 ± 2.9	0.943
Uric Acid (mg/dL)	–	–	6.8 ± 0.3	7.3 ± 0.3	7.8 ± 0.5	0.005
Cholesterol (mg/dL)	–	–	177.1 ± 7.1	181.9 ± 7.7	175.3 ± 10.6	0.504
Triglyceride (mg/dL)	–	–	149.7 ± 16.8	142.6 ± 13.8	134.4 ± 18.9	0.477
Low-density lipoprotein (mg/dL)	103.7 ± 1.9	104.0 ± 2.2	102.6 ± 5.5	105.3 ± 5.8	100.3 ± 7.6	0.65
Fasting glucose (mg/dL)	–	–	120.2 ± 7.6	119.2 ± 8.7	116.6 ± 8.5	0.855
Glycated hemoglobin (%)	6.7 ± 0.2	6.5 ± 0.9	7.0 ± 0.9	6.7 ± 0.2	7.7 ± 1.8	0.008
Dialysis treatment (%)	107 (10.3%)	48 (6.9%)	2 (1.8%)	17 (11.9%)	40 (45.5%)	<0.001
No. of follow-up measurements	3.3 ± 0.1	3.0 ± 0.1	4.2 ± 0.7	3.7 ± 0.5	3.1 ± 0.6	<0.001
Following time (days)	637.6 ± 37.9	680.6 ± 50.7	641.1 ± 102	542.6 ± 69.6	445.6 ± 85.6	<0.001
Comorbidity (%)						
Diabetes	588 (56.4%)	405 (57.9%)	588 (56.4%)	52 (46.9%)	84 (58.7%)	0.147
Hypertension	582 (55.9%)	332 (47.4%)	582 (55.9%)	84 (75.7%)	106 (74.1%)	<0.001
Cardiovascular diseases	200 (19.2%)	176 (25.1%)	200 (19.2%)	9 (8.1%)	11 (7.7%)	<0.001

Note: eGFR = estimated glomerular filtration rate.

**Table 2:** Crude hazard ratios for the effects on the risk of dialysis treatment.

Variables	Stage 3a			Stage 3b–5		
	*n* = 700			*n* = 342		
	Hazard Ratio	95% C.I.	*p*-Value	Hazard Ratio	95% C.I.	*p*-Value
Height (cm)	1.003	0.971–1.036	0.843	0.981	0.952–1.011	0.201
Weight (kg)	0.984	0.960–1.009	0.218	1.003	0.981–1.025	0.819
Age (years)	0.986	0.962–1.010	0.255	0.971	0.953–0.988	0.001
Male (%)	1.338	0.722–2.477	0.355	0.766	0.451–1.302	0.325
eGFR (mL/min/1.73 m^2^)	0.898	0.863–0.935	<0.001	0.878	0.849–0.908	<0.001
Hemoglobin (g/dL)	–	–	–	0.64	0.560–0.731	<0.001
Hematocrit (%)	–	–	–	0.926	0.903–0.951	<0.001
Serum albumin (g/dL)	–	–	–	0.199	0.136–0.294	<0.001
Serum creatinine (mg/dL)	27.7	8.275–92.720	<0.001	1.142	1.102–1.184	<0.001
Urea nitrogen (mg/dL)	–	–	–	1.031	1.024–1.038	<0.001
Sodium (mEQ/L)	–	–	–	0.934	0.874–0.998	0.043
Potassium (mEQ/L)	–	–	–	0.944	0.695–1.281	0.71
Calcium (mg/dL)	–	–	–	0.741	0.629–0.874	<0.001
Phosphate (mg/dL)	–	–	–	1.733	1.540–1.952	<0.001
ln urine protein/creatinine	1.699	1.347–2.142	<0.001	1.939	1.594–2.359	<0.001
Systolic blood pressure (mmHg)	1.007	0.991–1.022	0.394	1.011	0.999–1.022	0.074
Diastolic blood pressure (mmHg)	0.983	0.957–1.010	0.222	1.014	0.996–1.031	0.127
Uric Acid (mg/dL)	–	–	–	1.044	0.920–1.186	0.502
Cholesterol (mg/dL)	–	–	–	1.004	0.998–1.010	0.192
Triglyceride (mg/dL)	–	–	–	1.003	1.000–1.005	0.039
Low-density lipoprotein (mg/dL)	0.97	0.923–1.015	0.197	1.001	0.993–1.009	0.842
Fasting glucose (mg/dL)	–	–	–	1.001	0.995–1.007	0.687
Glycated hemoglobin (%)	1.258	1.007–1.572	0.044	1.035	1.008–1.062	0.011
Comorbidity (vs None)						
Diabetes	0.869	0.489–1.540	0.631	1.683	0.991–2.859	0.054
Hypertension	1.454	0.774–2.733	0.244	1.458	0.757–2.809	0.259
Cardiovascular diseases	0.77	0.343–1.728	0.526	0.465	0.113–1.908	0.287

Note: eGFR= estimated glomerular filtration rate; CI = confidence interval.

**Table 3:** Adjusted hazard ratios (AHR) for the effects and goodness of fit of the variables on the risk of dialysis treatment in the baseline model.

Variables	Stage 3a				Stage 3b–5							
	*n* = 700				*n* = 342							
	Model 1		Model 2		Model 1		Model 2		Model 3		Model 4	
	AHR	95% CI	AHR	95% CI	AHR	95% CI	AHR	95% CI	AHR	95% CI	AHR	95% CI
Age (year)	–	–	–	–	–	–	**0.974**	**0.953–0.996**	0.974	0.953–0.996	0.973	0.950–0.996
eGFR (mL/min/1.73 m^2^)	**0.938**	**0.888–0.992**	0.94	0.889–0.994	0.931	0.885–0.980	**0.926**	**0.879–0.975**	0.927	0.880–0.976	0.923	0.873–0.975
Hemoglobin (g/dL)	–	–	–	–	0.889	0.720–1.097	**0.861**	**0.698–1.063**	0.859	0.695–1.061	0.799	0.641–0.997
Serum albumin (g/dL)	–	–	–	–	0.507	0.287–0.897	**0.486**	**0.276–0.857**	0.485	0.275–0.855	0.461	0.263–0.809
Serum creatinine (mg/dL)	**4.437**	**0.856–22.99**	4.408	0.857–22.67	1.103	1.018–1.195	**1.094**	**1.002–1.196**	1.099	1.004–1.204	1.068	0.933–1.222
Urea nitrogen (mg/dL)	–	–	–	–	0.995	0.981–1.011	**0.999**	**0.984–1.015**	0.999	0.984–1.015	1	0.984–1.017
Sodium (mEQ/L)	–	–	–	–	0.977	0.918–1.041	**0.997**	**0.932–1.067**	0.998	0.933–1.067	1.011	0.942–1.085
Calcium (mg/dL)	–	–	–	–	0.798	0.499–1.276	**0.844**	**0.511–1.393**	0.846	0.515–1.389	0.844	0.535–1.330
Phosphate (mg/dL)	–	–	–	–	1.243	0.943–1.639	**1.122**	**0.833–1.512**	1.115	0.825–1.507	1.087	0.780–1.514
ln urine protein/creatinine	**1.586**	**1.258–1.999**	1.573	1.241–1.995	1.611	1.271–2.042	**1.485**	**1.184–1.864**	1.507	1.183–1.922	1.422	1.119–1.808
Glycated hemoglobin (%)	–	–	1.035	0.814–1.316	–	–	–	–	–	–	1.015	0.967–1.066
Systolic blood pressure (mmHg)	–	–	–	–	–	–	–	–	–	–	1.016	1.001–1.032
Triglyceride (mg/dL)	–	–	–	–	–	–	–	–	–	–	1.001	0.998–1.032
Diabetes (vs. None)	–	–	–	–	–	–	–	–	0.889	0.498–1.586	0.99	0.554–1.770
C statistics (*p*-value)	**0.786 (<0.001)**		0.783 (<0.001)		0.901 (<0.001)		**0.905 (<0.001)**		0.905 (<0.001)		0.905 (<0.001)	
Likelihood ratio	**49.85**		49.92		142		**147.4**		147.6		153.2	
*p*-value for the difference of likelihood ratio compared with that in the previous model)	–		0.783		–		**0.02**		0.905		0.133	

Note: eGFR = estimated glomerular filtration rate; CI = confidence interval. Models 1 to 4 are models including available variables of laboratory variables associated with CKD severity, demographics, comorbid conditions, and other underlying risk factors, such as biophysical and biochemical data, additively. Digits in bold indicate the optimal models.

**Table 4:** Adjusted hazard ratios (AHR) for the effects and goodness of fit of the variables associated with the risk of dialysis treatment in the dynamic model.

Variables	Stage 3a				Stage 3b–5							
	*n* = 700				*n* = 342							
	Model 1		Model 2		Model 1		Model 2		Model 3		Model 4	
	AHR	95% CI	AHR	95% CI	AHR	95% CI	AHR	95% CI	AHR	95% CI	AHR	95% CI
Age (year)	–	–	–	–	**–**	**–**	0.99	0.979–1.002	0.99	0.979–1.002	0.99	0.978–1.001
eGFR (mL/min/1.73 m^2^)	**0.932**	**0.911–0.953**	0.931	0.911–0.952	**0.896**	**0.869–0.923**	0.898	0.871–0.926	0.898	0.872–0.926	0.9	0.873–0.928
Hemoglobin (g/dL)	–	–	–	–	**0.927**	**0.837–1.026**	0.913	0.822–1.013	0.911	0.821–1.011	0.9	0.808–1.000
Serum albumin (g/dL)	–	–	–	–	**0.917**	**0.637–1.320**	0.874	0.606–1.261	0.884	0.613–1.275	0.991	0.980–1.003
Serum creatinine (mg/dL)	**0.919**	**0.77–1.097**	0.916	0.759–1.107	**1.004**	**0.943–1.068**	1.002	0.938–1.071	1.002	0.939–1.070	1.011	0.924–1.106
Urea nitrogen (mg/dL)	–	–	–	–	**0.994**	**0.987–1.000**	0.994	0.988–1.000	0.994	0.988–1.000	0.994	0.987–1.001
Sodium (mEQ/L)	–	–	–	–	**1.025**	**0.991–1.060**	1.027	0.993–1.062	1.029	0.995–1.064	1.029	0.994–1.065
Calcium (mg/dL)	–	–	–	–	**0.695**	**0.547–0.883**	0.731	0.572–0.935	0.74	0.577–0.948	0.768	0.599–0.985
Phosphate (mg/dL)	–	–	–	–	**1.106**	**0.953–1.284**	1.096	0.943–1.273	1.091	0.94–1.267	1.075	0.924–1.251
ln urine protein/creatinine	**1.715**	**1.462–2.012**	1.779	1.501–2.109	**1.114**	**1.029–1.207**	1.099	1.014–1.192	1.099	1.014–1.191	1.081	0.996–1.174
Glycated hemoglobin (%)	–	–	0.931	0.830–1.044	**–**	**–**	–	–	–	–	0.989	0.955–1.025
Systolic blood pressure (mmHg)	–	–	–	–	**–**	**–**	–	–	–	–	0.998	0.990–1.006
Triglyceride (mg/dL)	–	–	–	–	**–**	**–**	–	–	–	–	1.001	0.999–1.003
Diabetes (vs. None)	–	–	–	–	**–**	**–**	–	–	1.115	0.821–1.515	1.144	0.838–1.561
C statistics (*p*-value)	**0.772 (<0.001)**		0.776 (<0.001)		**0.822 (<0.001)**		0.824 (<0.001)		0.825 (<0.001)		0.827 (<0.001)	
Likelihood ratio	**125.6**		127.2		**279.5**		282.3		282.8		285.6	
*p*-value for the difference of likelihood ratio compared with that in the previous model	–		0.26		**–**		0.094		0.48		0.423	

Note: eGFR = estimated glomerular filtration rate; CI = confidence interval. Models 1 to 4 are models that include available laboratory variables associated with CKD severity, demographics, comorbid conditions, and other underlying risks, such as biophysical and biochemical data, additively. Digits in bold indicate the optimal models.

## Data Availability

The datasets generated and analyzed during the current study are not publicly available due to privacy/ethical restrictions but are available from the corresponding author on reasonable request.

## Supplementary Materials

The following are available online at https://www.mdpi.com/article/10.3390/jcm10143085/s1, Table S1: ICD-9-CM code for the two national program, Table S2: The collinearity of variables in the optimal baseline model, Figure S1: Schoenfeld residual plot of the optimal baseline model in Stage 3a CKD. Figure S2: Schoenfeld residual plot of the optimal baseline model in Stage 3b-5 CKD.
