# Supplementary material for: Static and Dynamic Prediction of Chronic Renal Disease Progression Using Longitudinal Clinical Data from Taiwan’s National Prevention Programs
Source: J Clin Med. 2021 Jul 13;10(14):3085. doi: 10.3390/jcm10143085 (PMC8306754; doi:10.3390/jcm10143085)
Supplement: Supplementary file 1 [file jcm-10-03085-s001.zip › jcm-1286897-supplementary.pdf]

Table S1. ICD-9-CM code for the two national program.

ICD-9-CM	Disease coding
NHI reimbursement plans that improve health care quality of early-stage chronic kidney disease	
016.0	Renal tuberculosis
095.4	Syphilis of kidney
189.0	Kidney, except pelvis
189.9	Urinary organ, site unspecified
223.0	Kidney, except pelvis
236.91	Kidney and ureter
250.4	Diabetes with renal manifestations
271.4	Renal glycosuria
274.1	Gouty nephropathy
283.11	Hemolytic-uremic syndrome
403	Hypertensive chronic kidney disease
404	Hypertensive heart and chronic kidney disease
440.1	Atherosclerosis of renal artery
442.1	Other aneurysm of renal artery
447.3	Hyperplasia of renal artery
572.4	Hepatorenal syndrome
580	Acute glomerulonephritis
581	Nephrotic syndrome
582	Chronic glomerulonephritis
583	Nephritis and nephropathy, not specified as acute or chronic
584	Acute renal failure
585	Chronic kidney disease (CKD)
586	Renal failure, unspecified
587	Renal sclerosis, unspecified
588	Disorders resulting from impaired renal function
591	Hydronephrosis
642.1	Hypertension secondary to renal disease, complicating pregnancy, childbirth, and the puerperium
646.2	Unspecified renal disease in pregnancy, without mention of hypertension
753.12	Polycystic kidney, unspecified type
753.13	Polycystic kidney, autosomal dominant
753.14	Polycystic kidney, autosomal recessive
753.15	Renal dysplasia
753.16	Medullary cystic kidney
753.17	Medullary sponge kidney
753.19	Other specified cystic kidney disease
753.2	Obstructive defects of renal pelvis and ureter
794.4	Kidney(Abnormal renal function test)
NHI Pre-ESRD patient care and education program	
585	Chronic kidney disease (CKD)
581	Nephrotic syndrome

Note: ICD-9-CM = the Ninth revision of the International Classification of Diseases; NHI= National Health Insurance; ESRD = end-stage renal disease.

Table S2. The collinearity of variables in the optimal baseline model.

Variable	Variance inflation factor
Stage 3a CKD	
eGFR	1.706
Creatinine	1.689
lnPCR	1.015
Stage 3b-5 CKD	
eGFR	2.672
Hemoglobin	1.921
Urea nitrogen	3.212
Creatinine	2.076
Sodium	1.092
Calcium	1.086
Phosphate	2.401
Albumin	1.684
lnPCR	1.446
Age	1.233

Note: eGFR= estimated glomerular filtration rate; lnPCR =ln urine protein/creatinine; CKD=chronic kidney disease.

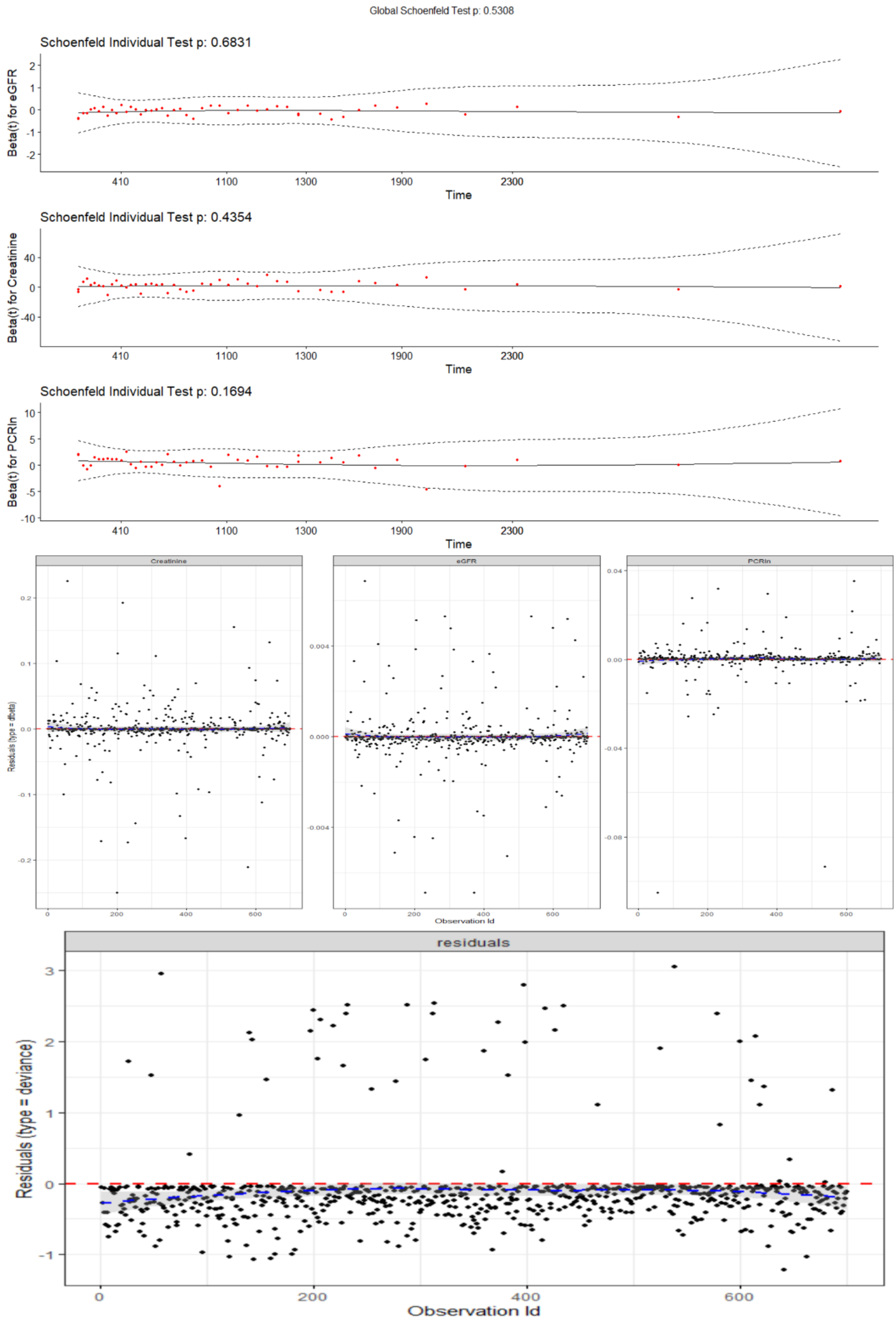

Figure S1. Schoenfeld residual plot of the optimal baseline model in Stage 3a CKD.

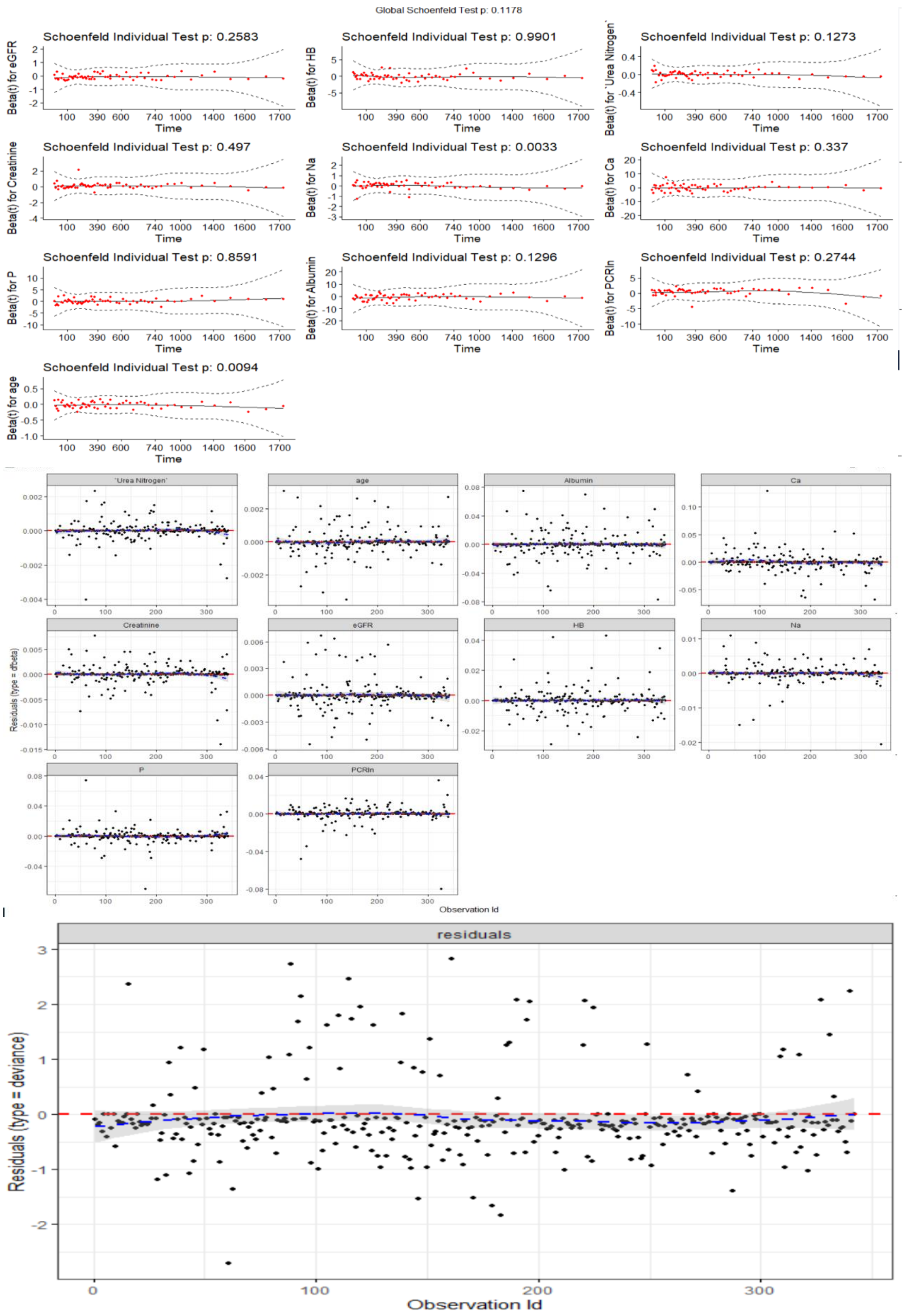

Figure S2. Schoenfeld residual plot of the optimal baseline model in Stage 3b-5 CKD.
